# microRNA-690 regulates induced pluripotent stem cells (iPSCs) differentiation into insulin-producing cells by targeting Sox9

**DOI:** 10.1186/s13287-019-1154-8

**Published:** 2019-02-15

**Authors:** Yang Xu, Yan Huang, Yibing Guo, Yicheng Xiong, Shajun Zhu, Liancheng Xu, Jingjing Lu, Xiaohong Li, Jian Wan, Yuhua Lu, Zhiwei Wang

**Affiliations:** 1 Department of Hepatobiliary and Pancreatic Surgery, Affiliated Hospital of Nantong University, Nantong, 226001 China; 2 Research Center of Clinical Medicine, Affiliated Hospital of Nantong University, Nantong, 226001 China; 3 Department of General Surgery, Affiliated Cancer Hospital of Nantong University, Nantong, 226361 China

**Keywords:** Insulin-producing cells (IPCs), Induced pluripotent stem cells (iPSCs), Differentiation, miRNA-690, Sox9, Wnt signaling pathway

## Abstract

**Background:**

The regulatory mechanism of insulin-producing cells (IPCs) differentiation from induced pluripotent stem cells (iPSCs) in vitro is very important in the phylogenetics of pancreatic islets, the molecular pathogenesis of diabetes, and the acquisition of high-quality pancreatic β-cells derived from stem cells for cell therapy.

**Methods:**

miPSCs were induced for IPCs differentiation. miRNA microarray assays were performed by using total RNA from our iPCs-derived IPCs containing undifferentiated iPSCs and iPSCs-derived IPCSs at day 4, day 14, and day 21 during step 3 to screen the differentially expressed miRNAs (DEmiRNAs) related to IPCs differentiation, and putative target genes of DEmiRNAs were predicted by bioinformatics analysis. miR-690 was selected for further research, and MPCs were transfected by miR-690-agomir to confirm whether it was involved in the regulation of IPCs differentiation in iPSCs. Quantitative Real-Time PCR (qRT-PCR), Western blotting, and immunostaining assays were performed to examine the pancreatic function of IPCs at mRNA and protein level respectively. Flow cytometry and ELISA were performed to detect differentiation efficiency and insulin content and secretion from iPSCs-derived IPCs in response to stimulation at different concentration of glucose. The targeting of the 3′-untranslated region of Sox9 by miR-690 was examined by luciferase assay.

**Results:**

We found that miR-690 was expressed dynamically during IPCs differentiation according to the miRNA array results and that overexpression of miR-690 significantly impaired the maturation and insulinogenesis of IPCs derived from iPSCs both in vitro and in vivo. Bioinformatic prediction and mechanistic analysis revealed that miR-690 plays a pivotal role during the differentiation of IPCs by directly targeting the transcription factor sex-determining region Y (SRY)-box9. Furthermore, downstream experiments indicated that miR-690 is likely to act as an inactivated regulator of the Wnt signaling pathway in this process.

**Conclusions:**

We discovered a previously unknown interaction between miR-690 and sox9 but also revealed a new regulatory signaling pathway of the miR-690/Sox9 axis during iPSCs-induced IPCs differentiation.

**Electronic supplementary material:**

The online version of this article (10.1186/s13287-019-1154-8) contains supplementary material, which is available to authorized users.

## Background

Type 1 diabetes (T1D) is defined as dysregulation of homeostatic control of blood glucose due to an absolute insulin deficiency caused by autoimmune destruction of insulin-secreting pancreatic β-cells [1]. The transplantation of β-cells from a pancreatic donor or augmentation of endogenous β-cells regeneration may lead to a cure for T1D. Unfortunately, these methods are restricted by donor tissue availability and tissue rejection and are thus far from being widely applied [2]. Insulin-producing cells (IPCs) derived from pluripotent stem cells in vitro may provide an alternative source of β-cells [3]; however, the rate of development of functional and mature IPCs is very low according to the present protocols [4], which will be improved by a thorough understanding of pancreatic organogenesis, including proliferation, differentiation, migration, and maturation of pancreatic progenitor cells.

Considerable evidence has verified that microRNAs (miRNAs) in pancreatic cells regulate gene expression through post-transcriptional modulation [5, 6]. Recently, the global influence of miRNAs on pancreatic development has been assessed by *Dicer*-knockout mouse embryos. *Dicer* deficiency resulted in alterations of islet architecture and differentiation markers, accompanied by enhanced apoptosis and defects in all types of endocrine cell formation, particularly that of β-cells [7]. Similarly, miR-375 is expressed specifically in pancreatic islets and regulates the proliferation and insulin secretion of β-cells by targeting myotrophin (MTPN) and phosphoinositide-dependent protein kinase-1 [8]. Knockdown of miR-375 in ob/ob mice led to a disproportionate ratio of β-cells to α-cells, high plasma glucagon levels, or even diabetes [9]. In addition, other miRNAs, such as miR-7 and miR-199b-5p, have been studied functionally and reported to selectively affect the development of pancreatic islets, promoting the proliferation of β-cells and miR-124a and regulating Foxa2 expression and intracellular signaling in β-cells [10–12]. These findings, as highlighted above, encouraged us to identify different layers of miRNA regulatory networks, which will provide greater insights into the roles of noncoding RNAs and help further elucidate β-cell biology, pancreas formation, and the molecular mechanisms of diabetes etiopathogenesis.

During pancreatic development, the sex-determining region Y (SRY)-box9 (Sox9) factor, which is known to function in campomelic dysplasia, XY sex reversal, and skeletal malformations, has been linked to the proliferation and differentiation of endocrine progenitors [13, 14]. Analysis of cases with Sox9 loss in pancreatic progenitor cells demonstrated a proportional reduction in FoxA2 and Onecut1 expression, along with upregulation of Hnf1b (TCF2), which resulted in a dramatic decrease in endocrine cells without changes in exocrine compartments [15]. Despite a fair understanding of the molecular mechanism by which Sox9 controls pancreatic development, only a few pathways regulated by Sox9 are known. Wnt/β-catenin signaling (WNT) has been demonstrated to participate broadly in the differentiation of stem cells, showing a negative regulatory relationship with Sox9 in various contexts [16, 17]. Furthermore, both CTNNB1 (β-catenin) and pGSK3β act as downstream target genes, increasing transcriptional activity and decreasing degradation by overexpression of Sox9 [14].

In this study, we identified miR-690 as a differentially expressed transcript during induced pluripotent stem cell (iPSCs)-induced IPCs differentiation in vitro. Surprisingly, predicted mRNA targets, such as Sox9, CTNNB1 (β-Catenin), and Stat3, were found to be crucial during the specification of pancreatic progenitor cells and terminal maturation of endocrine cells. Furthermore, the augmentation of miR-690 destabilized IPCs differentiation through direct binding to Sox9 and was likely to have a repressive effect on the Wnt pathway, suggesting an unreported role of miR-690 in modulating key transcription factors and signaling pathways.

## Materials and methods

### Animals

C57BL/6J mice were from the animal center of Nantong University. All animal experiments were performed according to the Institutional Animal Care guidelines and were approved by the Animal Ethics Committee of the Medical School of Nantong University.

### Cell culture and differentiation

Mouse GFP-iPSCs were obtained from the Innovative Cellular Therapeutics, Ltd. (Shanghai, China), maintained on feeders in mESC culture conditions, and induced to differentiate into pancreatic IPCs via a three-step protocol as previously described.

### RNA extraction and quantitative RT-PCR analysis

Total RNA was isolated using RNAiso Plus (TaKaRa). The first-strand cDNA synthesis for miRNA was performed by using the RevertAid First Strand cDNA Synthesis Kit (Thermo Scientific) and following the manufacturer’s instructions. The relative expression levels of each miRNA and mRNA were calculated by the 2^−ΔΔCt^ method as previously described, and GAPDH and U6 were used as the internal normalization controls. Each experiment was performed independently and repeated three times. The qRT-PCR primer sequences were designed and synthesized by GenScript Biotech Corp. (Nanjing, China).

### miRNA microarray assay and bioinformatic analysis of target genes

miRNA profiling of iPSC-derived IPCs was carried out by the Professional Oebiotech Corporation (Shanghai, China). In brief, total extracted RNA was labeled with the Agilent miRNA Complete Labeling and Hyb kit (Agilent, Santa Clara, CA, USA) and hybridized to an Agilent Mouse microRNA microarray V21.0. Then, a Gene Expression Wash Buffer kit (Agilent) was used to wash the microarray. Differentially expressed miRNAs (DEmiRNAs) were identified using GeneSpring software (version 13.1, Agilent Technologies, fold change ≥ 1.5, *P* value ≤ 0.05). TargetScan and microRNA.org were used to select target genes of DEmiRNAs (*P* ≤ 0.05 for both gene ontology (GO) and Kyoto Encyclopedia of Genes and Genomes (KEGG) analysis). The feasible regulatory relationships between miRNAs and target genes were analyzed using Cytoscape software (http://www.cytoscape.org/).

### Western blotting

Cells were washed with PBS and lysed on ice for 30 min with RIPA buffer (high) (Solarbio). Protein concentrations were detected using the BCA Protein Assay (Thermo Fisher Scientific). Total proteins were separated by SDS-PAGE, blotted on PVDF membranes (Millipore, Bedford, MA, USA), and probed with primary antibody in Antibody Dilution Buffer (Solarbio) at 4 °C overnight. After three washes in TBST, the membranes were incubated with HRP-conjugated secondary antibodies for visualization. Primary antibodies and HRP-conjugated secondary antibodies are listed: anti-*Sox9* antibody (Abcam), anti-beta catenin antibody (Abcam), anti-beta actin antibody as a loading control (Abcam), anti-phospho-GSK-3β (Ser9) rabbit mAb (Cell Signaling Technology), anti-phospho-CyclinD1 (Ser90) antibody (affinity), and goat anti-rabbit HRP antibody (affinity).

### Glucose-stimulated insulin secretion

iPSCs-derived IPCs were transferred into new 24-well plates for 12 h. After preincubation in Krebs-Ringer bicarbonate buffer (KRB) without glucose for 120 min, the cells were stimulated with KRB containing 0, 5, 15, 30, and 45 mM glucose for 120 min. The supernatant was collected. Insulin content and secretion from iPSC-derived IPCs were assessed by ELISAs, which were carried out using an ultrasensitive mouse insulin assay kit (Mercodia) following the manufacturer’s instructions.

### Immunofluorescence

iPSCs-derived IPCs grown on glass coverslips were washed with PBS and fixed with 4% paraformaldehyde for 15 min at room temperature. Then, these cells were washed thrice (10 min every time) and permeabilized with 0.5% (*v*/*v*) Triton X-100 for 15 min at room temperature. Next, 5% donkey serum was added for 60 min, and the cells were stained with different primary antibodies at 4 °C overnight. Then, the cells were stained with fluorescence secondary antibodies for 1 h and DAPI (Solarbio) for 15 min. Images were acquired using a Zeiss LSM 510 META confocal microscope (Carl Zeiss, Ltd.). Primary antibodies are listed as follows: anti-insulin antibody (Abcam), anti-C-peptide antibody (Abcam), anti-PDX1 antibody (Abcam), anti-SOX9 antibody (Abcam), antibody-beta catenin antibody (Abcam), anti-NKX6.1 (D804R) rabbit mAb (Cell Signaling Technology). Secondary antibodies included donkey anti-rabbit (Alexa Fluor® 647, Abcam), donkey anti-rabbit (Alexa Fluor® 555, Abcam), goat anti-guinea pig (Alexa Fluor® 647, Abcam), donkey F(ab,)2 anti-goat (Alexa Fluor® 594, Abcam), and donkey anti-goat (Alexa Fluor® 647, Abcam) antibodies.

### Flow cytometry

For identification of the insulin-positive population, 1 × 10^6^ iPSCs-derived IPCs were digested with trypsin, washed with PBS, and resuspended as single cells by incubation in Reagent 1: Fixation (Beckman Coulter) for 15 min. Then, the cells were washed once in PBS, incubated in Reagent 2: Permeabilization (Beckman Coulter) for 20 min, and washed once in PBS. Next, the cells were resuspended in PBS with primary antibody and incubated for 30 min. The cells were then washed with PBS twice and analyzed with the BD FACSCalibur system (BD Biosciences). The results were analyzed using FlowJo software. All procedures were carried out at room temperature. The primary antibody was anti-h/b/m insulin APC-conjugated rat IgG2A (R&D Systems). The isotype antibody was rat IgG2A control APC-conjugated.

### Dual-luciferase reporter assay

A luciferase reporter assay was performed to observe interactions between miR-690 and Sox9. Wild-type Sox9 and the mutant Sox9 were cloned into the Pezx-FR02 reporter vector for miR-690 targeting. Pezx-FR02 or Pezx-FR02-Sox9-MUT was co-transfected with miR-690 mimic or miRNA mimic control. Firefly and Renilla luciferase activities were assayed with a Dual-Luciferase Assay (Promega, Madison, USA) at 48 h post-transfection according to the manufacturer’s instructions.

### Statistical analysis

Data are presented as the mean ± standard deviation (SD) from at least three independent experiments. Significant differences in the relative miRNA or mRNA levels between the experimental groups and their negative controls were determined via Student’s *t* test using GraphPad Prism 7.0 (GraphPad Software, Inc.). A *P* value < 0.05 was considered significant.

## Results

### In vitro differentiation of IPCs

**Fig. 1:**
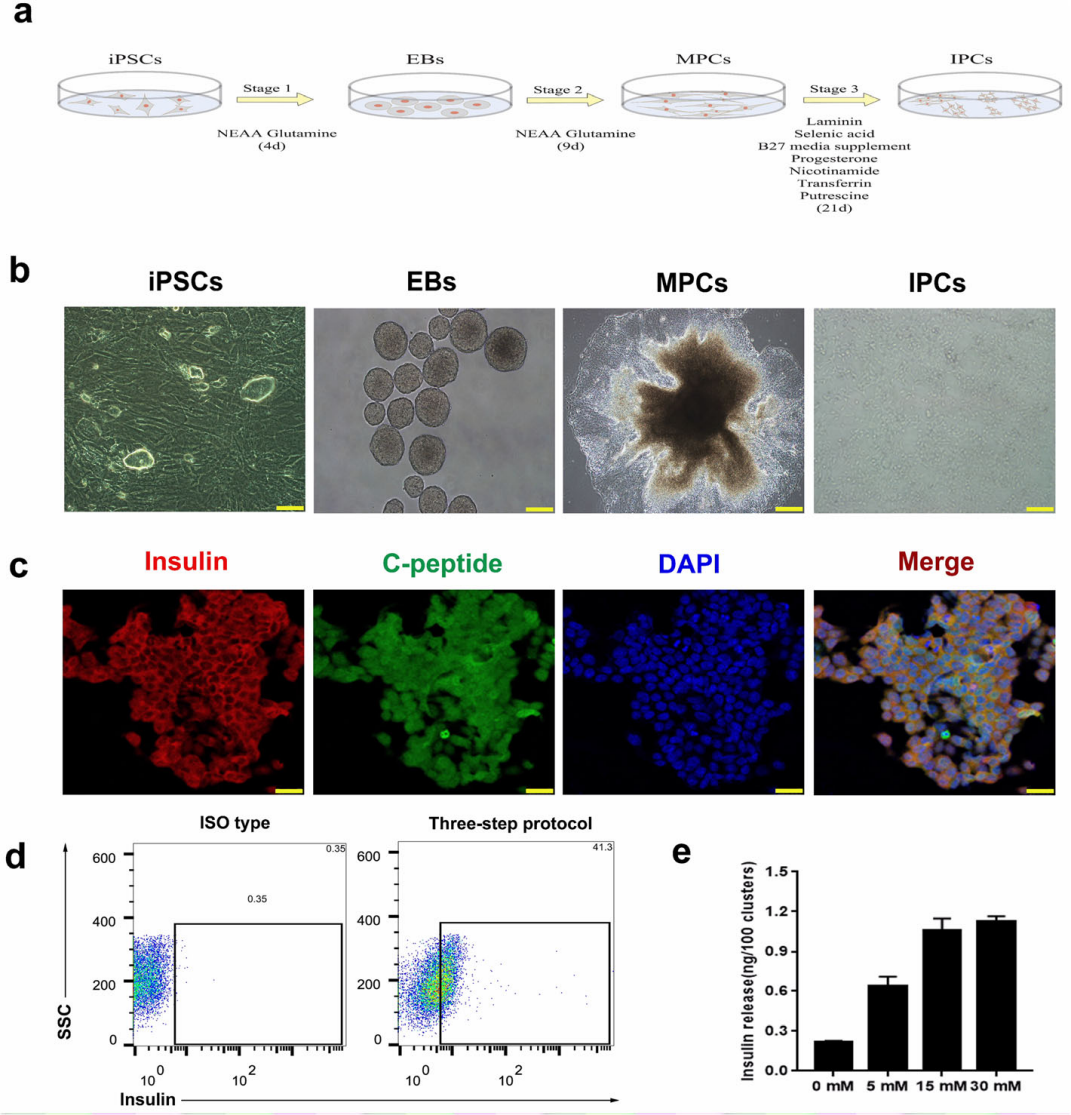
Overview of the differentiation protocol. **a** Summary of the three-step differentiation protocol. EBs embryoid bodies, MPs multilineage progenitor. **b** Morphologies of differentiating iPSCs into IPCs at different time points during differentiation. Scale bar: 20 μm. **c** Immunofluorescence assay of iPSCs at step 3 on day 21. Co-immunostaining of insulin (red) with C-peptide (green); nuclear DAPI staining is shown in blue. Scale bar: 75 μm. **d** Flow cytometry plots illustrating the protein expression of insulin in populations of iPSC-derived IPCs. Black text indicates the percentage of insulin. **e** Glucose-stimulated insulin secretion in vitro. iPSC-derived IPCs on day 21 of the three-step protocol were exposed to different glucose concentrations (0, 5, and 15 mM). The insulin concentration levels were determined

The differentiation protocol has been described by Huang et al. (Fig. 1a, b) [4, 18]. The iPSCs obtaining from the Innovative Cellular Therapeutics, Ltd., were identified (Additional file 1: Figure S1). Importantly, pancreatic β-cells are the only IPCs in humans and animals. C-peptide is the active form of insulin. We detected these two markers of mature β-cells in iPSC-derived IPCs on day 21 of step 3 to evaluate the efficiency of these insulin-secreting cells. Immunofluorescence assays showed that the majorities of the cells were positive for insulin and C-peptide (Fig. 1c). The flow cytometry results also showed that 41.3% ± 0.35% of iPSCs-derived IPCs at the final stage were insulin^+^ (Fig. 1d). To determine whether the differentiated cells respond to glucose stimulation, we assessed insulin secretion by exposing IPCs to glucose at different concentrations (0, 5, 15, and 30 mM). Treatment with glucose increased insulin secretion in these IPCs, with a peak at the 15 mM glucose concentration. No more insulin was induced when the glucose concentration increased to 30 mM, suggesting that these IPCs reached the upper limit of their insulin secretion capacity in response to glucose (Fig. 1e).

### miRNA profiling during IPCs differentiation

**Fig. 2:**
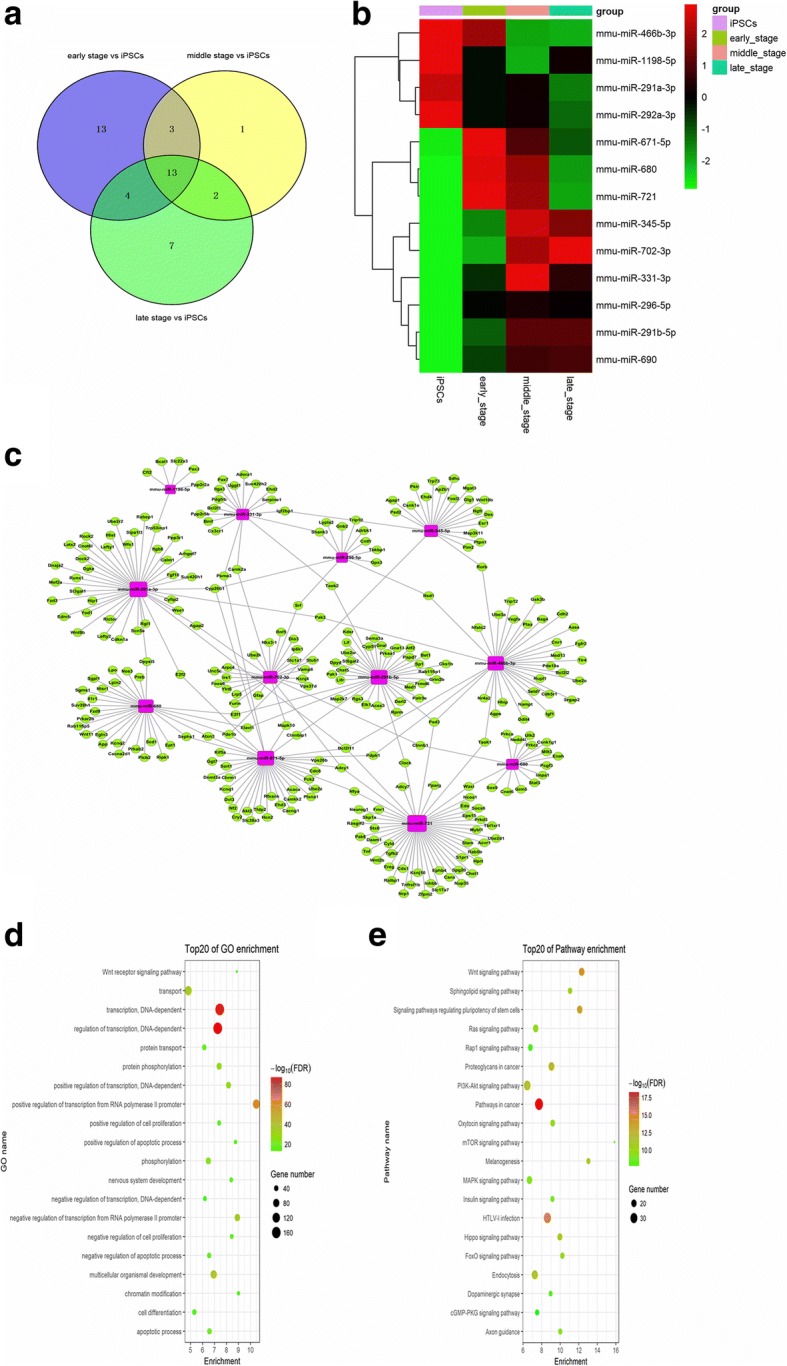
Differentially expressed miRNA profiling and bioinformatic analysis. Differentially expressed miRNAs (*P* < 0.05) were analyzed by hierarchical clustering of log_2_ values. **a** Venn diagram showing separate and overlapping differential expression of miRNAs during iPSCs-derived IPCs at the early, middle, and late stages of step 3 compared to that of iPSCs. **b** Heatmap shows selected differentially expressed miRNAs (fold change 1.5 and *P* value < 0.05). **c** The regulatory network of miRNA-target genes. Green circles represent target genes, and purple circles represent differentially expressed miRNAs. **d** Differentially expressed pathways were analyzed by gene ontology (GO) analysis. **e** KEGG pathway enrichment analysis for target genes. The size of the bubbles represents the number of target genes associated with each pathway

To screen the differentially expressed miRNAs (DEmiRNAs) related to IPCs differentiation, we performed miRNA microarray assays by using total RNA from our iPSCs-derived IPCs containing undifferentiated iPSCs and iPSCs-derived IPCs at day 4 (early stage), day 14 (middle stage), and day 21 (late stage) during step 3. A Venn diagram was used to compare several miRNAs differentially expressed during the three-step induction. The results showed that there were 13 common miRNAs during the three-step induction (Fig. 2a). The miRNA expression levels at different time points were clustered and are shown graphically (Fig. 2b).

To further understand the role of 13 common DEmiRNAs in iPSCs-derived IPCs, we performed the bioinformatics prediction analysis using two databases (TargetScan and miRanda) respectively to search for putative target genes. There were 332 common target genes after combining data predicted by two databases (miRanda threshold value: binding energy ≤ − 16.0, align score ≥ 158, TargetScan threshold value: context score percentile ≥ 30, data not shown). We explored the connections between the DEmiRNAs and putative target genes by building a regulatory network for miRNA-target genes using Cytoscape software (Fig. 2c). Then, we investigated the target genes in the KEGG pathways to further study the biological function of the DEmiRNAs (Fig. 2d). Interestingly, the WNT signaling pathway was located at the top of the 20 most enriched pathways. Our pathway analysis partly revealed the function of the signature miRNAs, and signal-related function was highlighted among all the subsystems, which was consistent with GO function analyses of the target genes (Fig. 2e). To verify the bioinformatic results, we performed qPCR, showing that miR-296, miR-331, miR-345, and miR-690 levels were consistent with the previous trends (Additional file 2: Figure S2). Of the transcripts that we identified, miR-690, which was persistently highly expressed in the full step 3, drew our attention, as it was reported to regulate *Runx2*-induced osteogenic differentiation of myogenic progenitor cells; these findings suggest that it may mediate organism differentiation and development. Then, we concentrated on the miR-690 functions during IPCs differentiation.

### Overexpression of miR-690 impaired IPCs in vitro

**Fig. 3:**
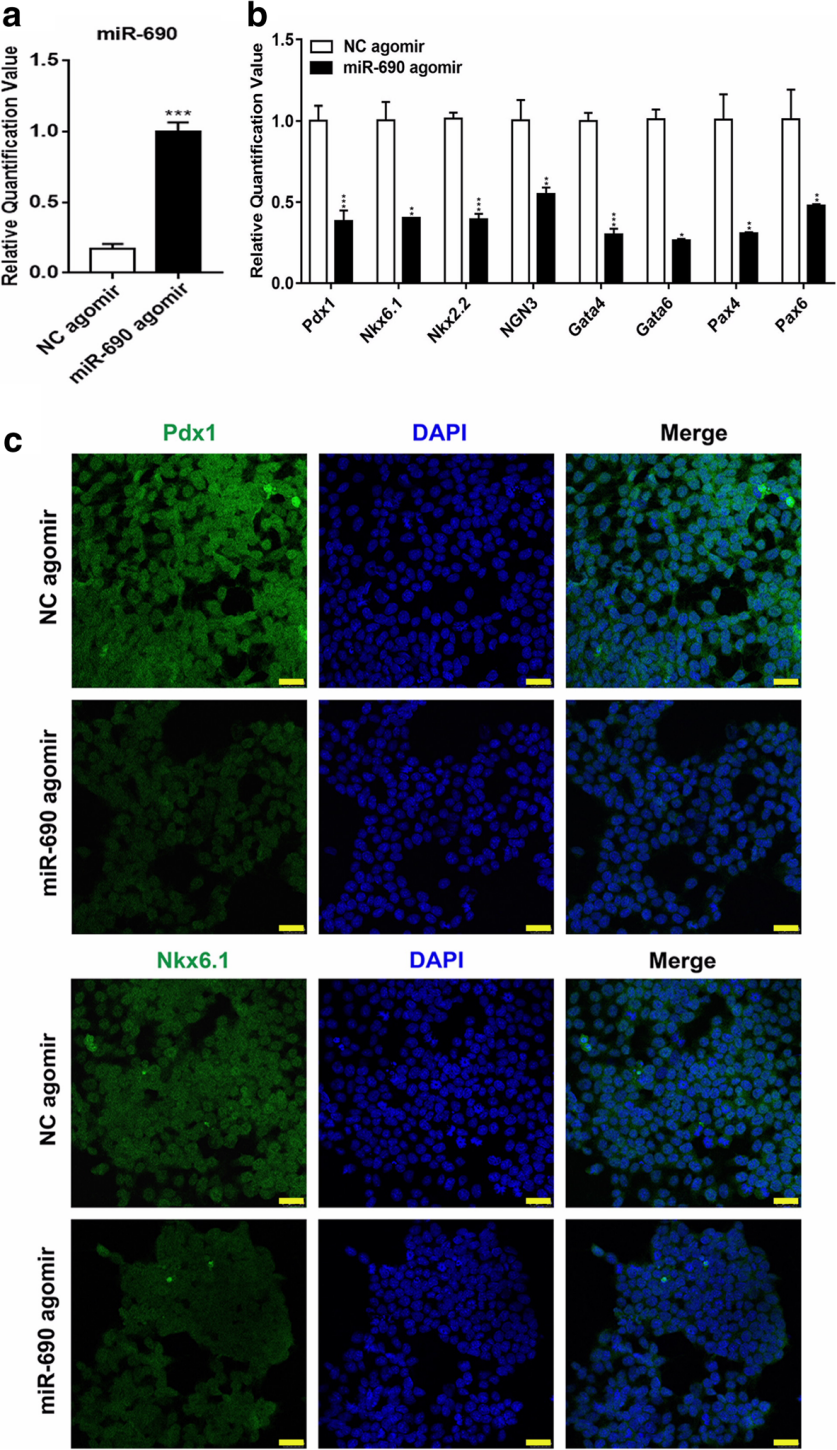
Overexpression of miR-690 inhibits pancreatic differentiation potential. This group of experiments tested the functions of iPSCs-derived IPCs on day 4 of the second step. Quantitative RT-PCR analysis of the expression levels of **a** miR-690 and **b** several key transcription factors during the development of pancreatic β-cells (Pdx1, NGN3, Nkx2.2, Nkx6.1, Gata4, Gata6, Pax4, Pax6). GAPDH was used as the internal control. Error bars show mean ± standard deviation (SD) (*n* = 3). *P < 0.05, **P < 0.01, ***P < 0.001, ****P < 0.0001. **c** Immunofluorescence assay (Nkx6.1 and Pdx1, green; nuclear, blue; scale bar 75 μm) for protein expression level of Nkx6.1 and Pdx1

**Fig. 4:**
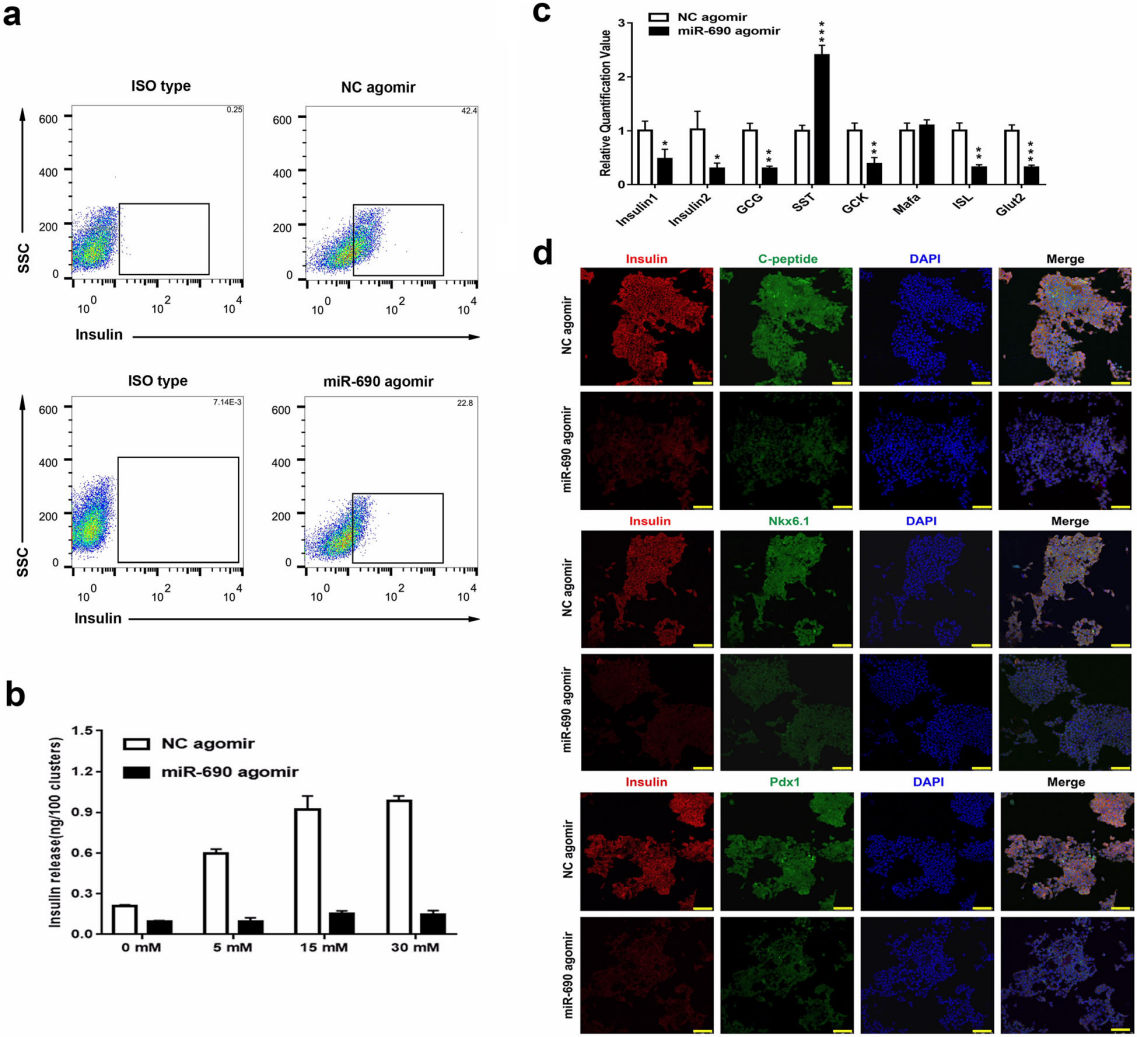
Overexpression of miR-690 impaired the functions of terminal iPSCs-derived IPCs. This group of experiments tested the functions of iPSCs-derived IPCs on day 21 of the second step. **a** Flow cytometry plots illustrating the protein expression of insulin in populations of iPSCs-derived IPCs. Black text indicates the percentage of insulin. **b** Glucose-stimulated insulin secretion in vitro. iPSCs-derived IPCs on day 21 of the third step were exposed to different glucose concentrations (0, 5, 15, and 45 mM). The insulin concentration levels were determined. **c** Quantitative RT-PCR analysis of the mRNA expression levels of key endocrine markers (insulin1, insulin2, GCG, SST, GCK, Mafa, ISL, Glut2). GAPDH was used as the internal control. Error bars show mean ± standard deviation (SD) (*n* = 3). *P < 0.05, **P < 0.01, ***P < 0.001. **d** Immunofluorescence assays of protein expression levels of some key markers. Co-immunostaining of insulin (red) with C-peptide (green), insulin (red) with Pdx1 (green), insulin with Nkx6.1 (green); nuclear DAPI staining is shown in blue. Scale bar 75 μm

To explore the specific function of miR-690 in the progression of the three-step induced differentiation, we constructed an agomir vector targeting miR-690 (miR-690-agomir), and miR-690 was overexpressed in MPCs on day 4. The overexpression efficiency of agomir-miR-690 was confirmed by qPCR analysis (Fig. 3a). Upregulated miR-690 in MPCs reduced the mRNA expression of several key transcription factors critical for early pancreatic development such as Pdx1, Ngn3, Nkx6.1, Gata4, and Pax4, although the deletion of these nonspecific factors alone was enough to abrogate pancreatic lineage induction (Fig. 3b). Immunostaining assays partially verified the results of quantitative RT-PCR analysis (Fig. 3c). As expected, IPCs overexpressing miR-690 showed a weak response to glucose stimulation, and high expression of these markers was correlated with the maturation of β-cells. Moreover, flow cytometry showed that the population of insulin^*+*^ cells significantly decreased from 42.4% ± 0.25% to 22.8% ± 0.007% from cells with NC-agomir compared to cells with miR-690-agomir (Fig. 4a). The ELISA results of mature IPCs (late stage/day 21) showed that insulin secretion decreased after glucose stimulation (Fig. 4b), indicating that IPCs were unable to reduce their glucose concentrations compared with NC cells. Also, we found that IPCs generated after overexpression of miR-690 showed significantly lower mRNA levels of mature β-cell and mature α-cell markers, such as insulin 1, insulin 2, *ISL*, *GCK*, and *GCG*, than NC-agomir-transfected cells on day 21 of the late stage through quantitative RT-PCR analysis. Interestingly, *SST* expression of δ-cells was opposite to that of mature β-cells and mature α-cells, and Mafa expression showed no significant difference between the two groups of cells (Fig. 4c). In addition, immunostaining assays confirmed that the co-expression of insulin/C-peptide, insulin/Nkx6.1, and insulin/Pdx1 was consistent with the results from previous quantitative RT-PCR assays (Fig. 4d). All these findings showed that miR-690 suppressed the maturation and endocrine functions of IPCs derived from iPSCs, indicating that miR-690 might be a critical regulator of β-cells differentiation.

### miR-690 inhibits Sox9 by targeting its 3′ untranslated region (UTR)

**Fig. 5:**
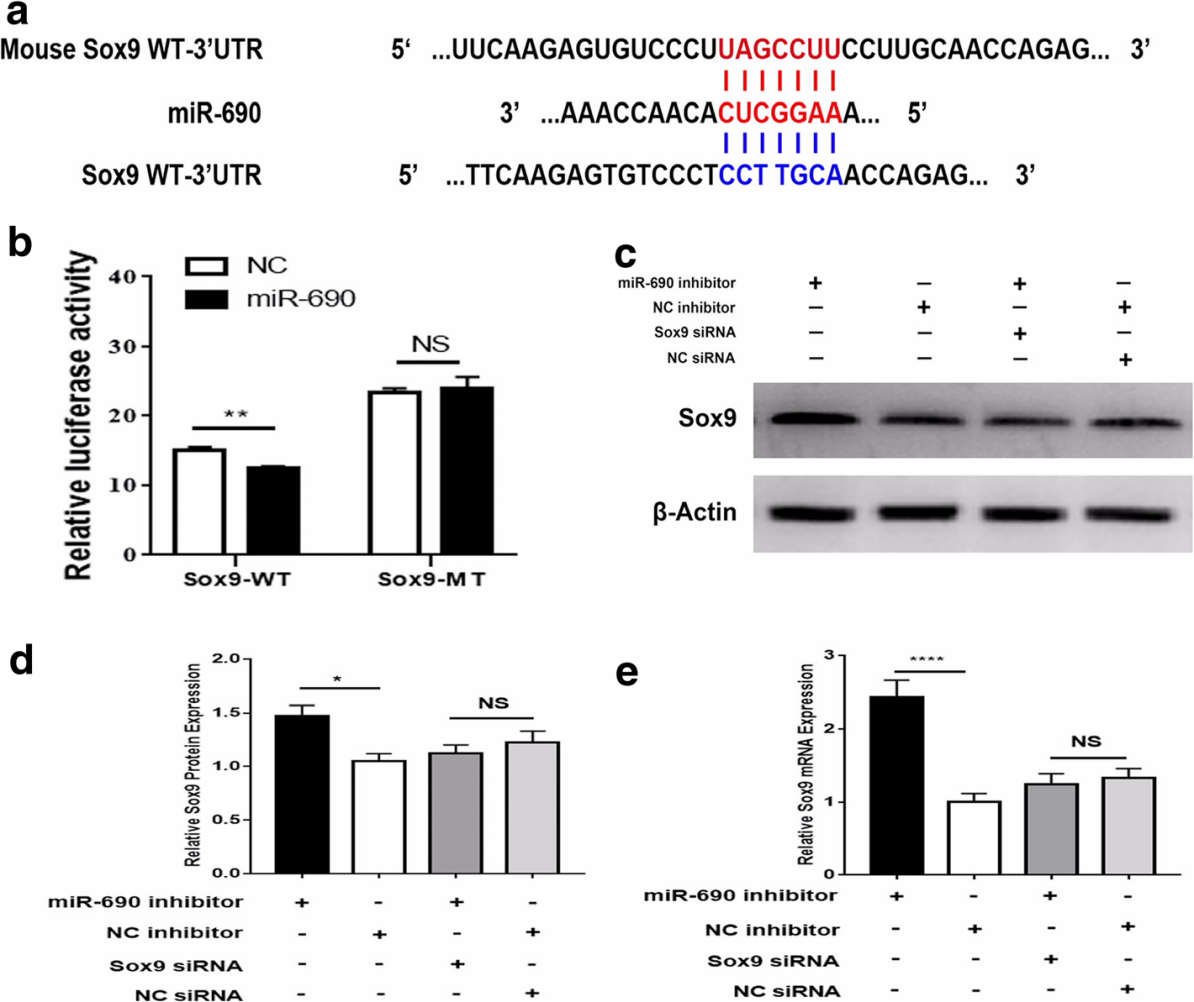
miR-690 directly targeted Sox9 in iPSCs-derived IPCs. **a** Predicted miR-690 targeting sequence in the 3′UTR of Sox9 (Sox9 WT-3′UTR) and the mutant form of the Sox9 3′UTR (Sox9 MUT-3′UTR). **b** Dual-luciferase reporter assays to determine the influence of miR-690 on Sox9 3′UTR activity in iPSCs-derived IPCs. Data are the mean ± SD of three independent assays. **c**–**e** Quantitative RT-PCR and Western blotting analyses of the effects of miR-690 knockdown (miR-690 inhibitor) on the expression level of Sox9 and the effects of miR-690 knockdown (miR-690) on the repressive effects of Sox9 knockdown (Sox9 siRNA). β-Actin was used as the loading control. GAPDH was used as the internal control for mRNA. Error bars show the SD (*n* = 3)

To further dissect the molecular mechanism of the inhibitory effect of miR-690 on IPCs differentiation, we performed the bioinformatics prediction analysis by using TargetScan and miRanda and combined with the results from RNA-seq (Huang, et al.) to predict the target genes of DEmiRNAs. miR-690 has 15 putative target genes (Prkca, Nedd4l, Ulk2, Prkcz, Csnk1g1, Mllt3, Enah, Pcgf3, Impa1, Stat3, Grm5, Cnot6, Sox9, Wasl, and Ctnnb1). Then, we built a regulatory network to show the connections between DEmiRNAs and target genes (Fig. 2c). Among these predicted genes, *Sox9*, a marker of pancreatic progenitor cells, and the genes encoding key transcription factors for the development of β-cells were notable. Next, we performed a dual-luciferase reporter assay to experimentally determine whether miR-690 targeted Sox9 directly. We transfected HEK293T cells with a luciferase plasmid containing the wild-type 3′ UTRs of Sox9 or its mutant version downstream of the firefly luciferase cDNA in the pEZX-FR02 vector (Fig. 5a). The results showed that the co-transfection of miR-690 mimics into 293 T cells led to a decrease of up to 83% in luciferase activity by miR-690 but had nearly no effect on the mutant reporter activity (Fig. 5b). Furthermore, knockdown of miR-690 reversed the repressive effects of siRNA-Sox9 on the mRNA and protein levels of Sox9 (Fig. 5c–e). These findings indicated that Sox9 was the authentic target of miR-690 in our induced IPCs.

### miR-690 is likely to negatively regulate β-cells differentiation by inactivating WNT signaling through Sox9

**Fig. 6:**
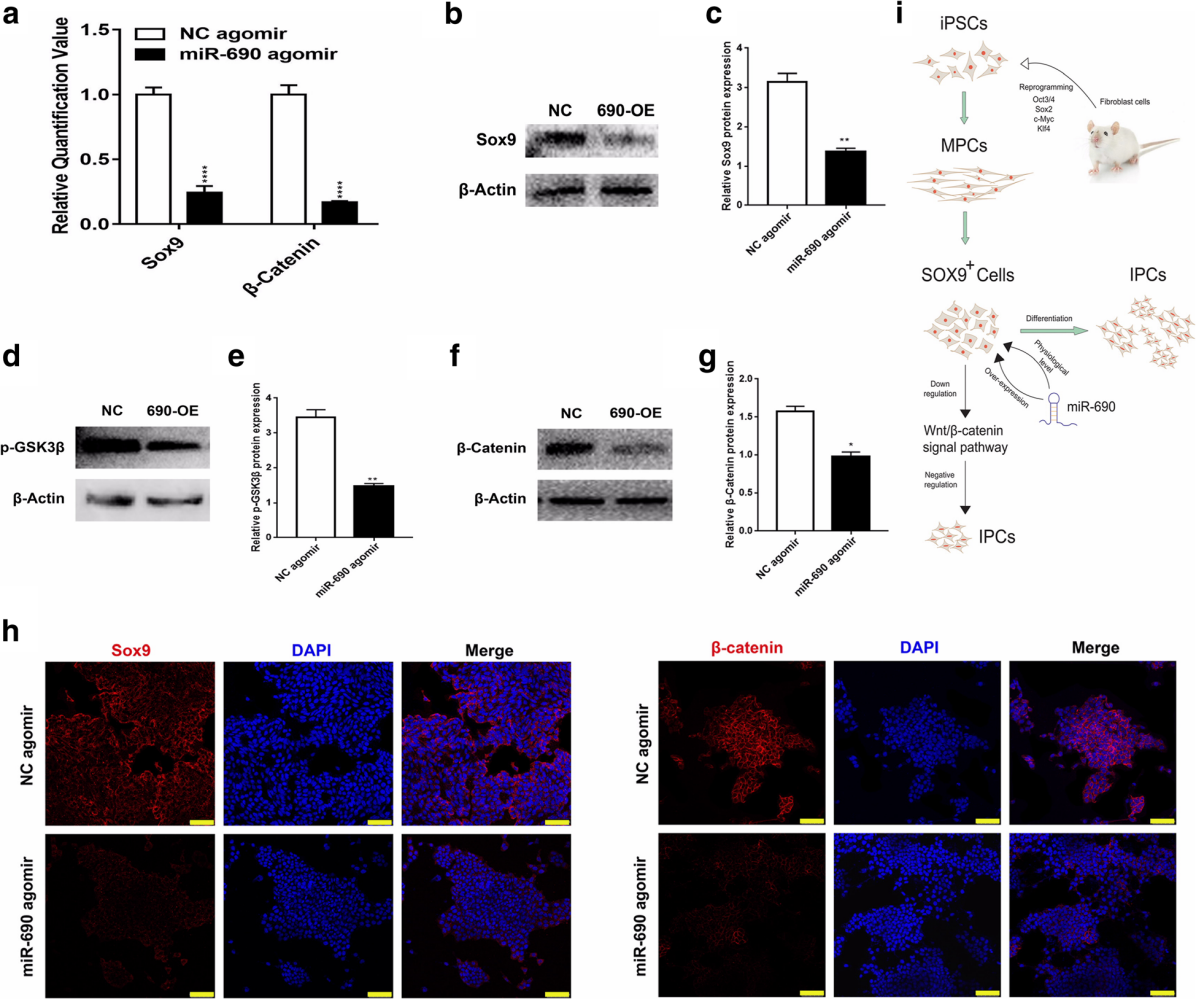
miR-690 may affect the differentiation of IPCs by inactivating the expression of the Wnt signaling pathway. **a** Quantitative RT-PCR analysis of the expression levels Sox9 and β-catenin. The scale bar represents 100 μm. Western blotting analysis of the effects of miR-690 overexpression on Sox9 (**b**, **c**), p-GSK3β (phosphorylated-GSK3β) (**d**, **e**), and β-catenin (**f**, **g**) (690-OE means 690 overexpression/miR-690 agomir). β-Actin was used as the loading control. **h** Immunofluorescence assay (Sox9 and β-catenin, red; nuclei, blue; scale bar 75 μm) of the protein level of Sox9 and β-catenin. **i** Schematic diagram of the supposed role of miR-690 in iPSCs-derived IPCs differentiation.

Sox9 has been reported to play a role in regulating Wnt signaling, which influences pancreatic development and modulates mature β-cell functions, such as insulin secretion, survival, and proliferation. Sox9 was chosen for further analysis in our study and validated by both qPCR analysis at the mRNA level and Western blot and immunostaining assays at the protein level (Fig. 6a–c and h). Because the phosphorylation and inactivation of GSK3-β may lead to activation and nuclear translocation of β-catenin, we detected the level of GSK3-β phosphorylation when miR-690 was overexpressed. As expected, a more than 1.5-fold decrease in phosphorylated GSK3-β and a more than 2-fold decrease in β-catenin activity were observed (Fig. 6d–h). We speculated that in our induced models, miR-690 may inactivate the WNT signaling pathway through Sox9 which will be the focus of our future research (Fig. 6i).

### Reversal of hyperglycemia after transplantation of IPCs into STZ-induced diabetic mice

**Fig. 7:**
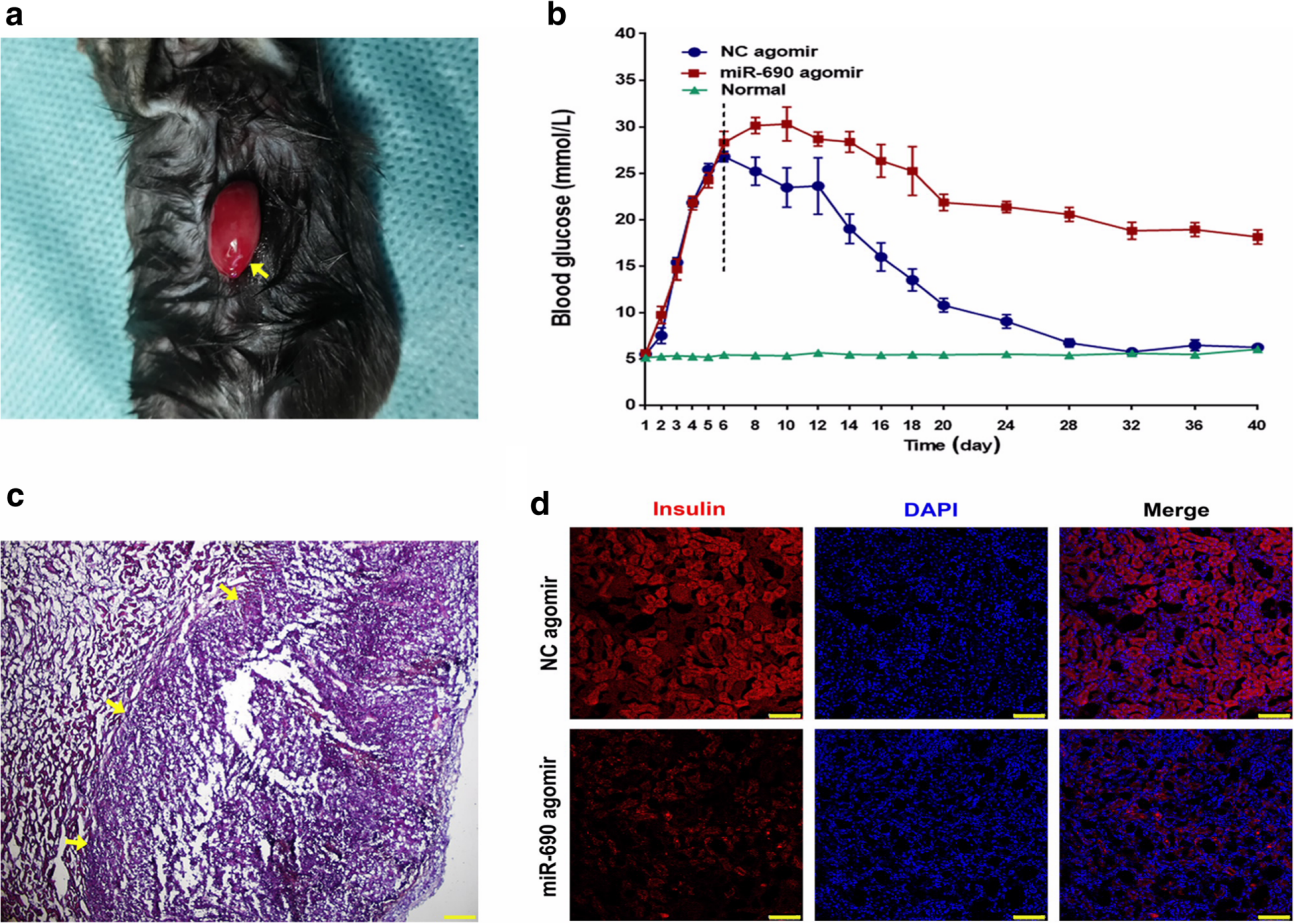
iPSCs-derived IPCs reverse diabetes in vivo. **a** Image of the entire kidney with iPSCs-derived IPCs engrafted under the kidney capsule and harvested at 25 days post-transplant. (~ 1 × 10^6^ cells/mouse, *n* = 6 /group). **b** Blood glucose levels were measured pre- and post-transplantation for over 30 days. **c** Hematoxylin and eosin (H&E) staining image of iPSCs-derived IPCs grafts in the kidney capsule 25 days after transplantation. Scale bar 200 μm. **d** Immunofluorescence staining of whole grafts for insulin (red); nuclear DAPI staining is shown in blue. Scale bar 75 μm

We next sought to explore whether miR-690 could modulate glucose homeostasis by transplanting miR-690-overexpressing IPCs and negative control cells into anemic capsule kidneys of mice treated with streptozotocin (STZ), which specifically destroys mouse β-cells (Fig. 7a). After transplantation, populations from the NC group needed nearly 28 days to reverse the hyperglycemia. Although the blood glucose level was decreased, mice transplanted with the miR-690 agomir still showed glycemia (Fig. 7b). Not surprisingly, the body weight of transplanted mice in the miR-690 overexpression group was significantly lower than that of the control group and healthy mice (data has not shown). At 40 days post-transplant, excised iPSCs-derived IPCs grafts were highly compact and homogenous and did not have regions of expanded ducts (Fig. 7c). Immunofluorescence staining revealed insulin-positive clusters of cells in the graft surrounded by connective tissue producing endocrine hormones (Fig. 7d).

## Discussion

iPSCs, which are derived from somatic cells, allow for the patient-specific functional β-cells in vitro that will free diabetic populations from daily insulin injections and prevent life-threatening complications, generate sufficient β-cells for transplantation, and also avert immune suppression to repress auto- and allo-immunity [1, 19]. Although many attempts have been made to acquire mature, glucose-responsive IPCs entirely in vitro, the results of these studies lacked convincing evidence [19]. Multiple core transcription factors, signaling pathways, and noncoding RNAs have been confirmed to be required for pancreatic β-cells differentiation potential in potent stem cells [10, 20–24]. Increasing evidence shows that miRNAs, as important epigenetic factors that regulate gene expression and determine cell fate in pancreatic β-cells, mediate β-cells biological activities, including differentiation, proliferation, apoptosis, and insulin secretion [6, 25]. However, the mechanisms of miRNAs in β-cells differentiation of iPSCs remain unknown.

This study adopted a three-step protocol mimicking normal pancreatic formation to screen for differentiation-associated miRNAs during iPSCs-induced IPCs differentiation in culture. According to the miRNA array analysis data, 13 miRNAs with markedly different expression levels were identified (Additional file 1: Figure S1), and we found that miR-690 was significantly upregulated in step 3 compared to that in iPSCs. To explore the specific function of miR-690 in IPCs differentiation, we overexpressed miR-690 in progenitor cells on day 4 of step 3 and found that pancreatic progenitor markers, such as Pdx1 and Sox9, and the early endocrine progenitors NGN3, Nkx6.1, and Pax4 were downregulated after 48 h. At the final stage of our protocol, miR-690 overexpression significantly impaired the maturation and endocrine function of IPCs (Fig. 3). However, the mRNA level of SST increased unexpectedly after miR-690 overexpression, suggesting that this miRNA may promote the differentiation of δ-cells.

To elaborate on the mechanism by which miR-690 regulates IPCs formation, we used bioinformatic analysis. Combined with the RNA-seq data detected previously, these results identified Sox9 as an underlying target gene of miR-690. Sox9 is widely known as a pancreatic progenitor marker that influences endocrine pancreatic development and modulation of mature β-cells functions [14]. The prevailing theory is that miRNAs regulate gene expression post-transcriptionally by binding to the 3′ untranslated sequence of the targeted mRNA to silence its corresponding target genes [26, 27]. Then, we demonstrated that Sox9 was a direct target of miR-690 using a luciferase reporter assay (Fig. 5). Furthermore, overexpression of miR-690 decreased the protein levels of Sox9 and β-catenin (Fig. 6), indicating that this noncoding RNA may regulate the Wnt signaling pathway, which has been thoroughly investigated and is necessary for controlling the development of β-cells and their function [16, 28, 29]. These findings suggested that the important function of miR-690 during IPCs differentiation was predominantly regulated by the miR-690/Sox9 and β-catenin axes, confirming that the interactions of miRNAs and transcription factors were involved in the differentiation of mouse iPSCs to IPCs. β-catenin is an important effector of the Wnt pathway [30]. To date, the role of Wnt signaling in pancreatic development has been disputed. The majority of studies have noted the primary role of Wnt signaling in the development of the exocrine compartments of the pancreas and confirmed that abolishment of the signaling pathway resulted in an almost complete lack of exocrine cells; however, the influence of Wnt signaling on endocrine cells, especially on pancreatic β-cell development, is still undefined [31]. Previous studies have reported that knockdown of the Sox9 gene in human islet epithelial cells significantly decreases the expression of phosphorylated GSK3-β at the protein level, leading to a prominent decline in the expression of cyclin D1 and other target genes of the Wnt signaling pathway [14]. Therefore, we examined the Wnt signaling activity by detecting the expression of p-GSK3-β. Interestingly, the results showed that miR-690 overexpression simultaneously decreased Sox9 and phosphorylated GSK3-β at the protein level. We speculated that miR-690 may mediate the Wnt signaling pathway via binding to Sox9 and lead to a decline in phosphorylation of GSK3-β and a decrease in β-catenin, which are the effectors of this pathway. Furthermore, other researchers have shown that pancreatic β-cells differentiation is complex and a result of the interaction of multiple signaling pathways, such as Notch, Fgf, Wnt, and others. Thus, the specific regulatory mechanism between miR-690 and the Wnt signaling pathway and whether other signaling pathways are regulated by miR-690 require further exploration.

Recently, miR-690 was reported to mediate osteogenic differentiation of human myogenic progenitor cells through its target NF-kappaB p65, indicating that miR-690 may play different roles in the development and differentiation of different organs and tissues [32]. Many studies have shown that Sox9 downregulation is important for early lineage bifurcation of endocrine progenitors and pancreatic β-cells development [15, 33–36]. In our study, the expression of miR-690 at an appropriate level is vital to the maturation and differentiation of IPCs. However, prematurely upregulated Sox9 resulted in deficient IPC differentiation in vitro, indicating that miR-690 activity may need to be within a narrow range to avoid detrimental consequences. Therefore, further exploration of the function of the miR-690/Sox9/Wnt signaling pathway in pancreatic β-cells differentiation, development, and maturation may be required to systematically uncover the critical function and mechanism of miR-690 in vitro and in vivo.

## Conclusion

We found that miR-690, a rarely studied noncoding RNA, played an important role in the differentiation of iPSCs-derived IPCs. MiR-690 regulates the expression of transcription factor Sox9 and may have an influence on Wnt signaling pathway in the differentiation process. These findings not only indicate that miR-690 mediates differentiation of iPSCs-derived IPCs through Sox9 and affects Wnt signaling pathway, but also provide novel evidence for the regulatory potential mechanisms of miRNAs in development associated with insulin-producing cells derived from induced pluripotent cells.

## Additional files

Additional file 1: **Figure S1.** Identification of iPSCs. a The GFP-iPSCs were purple which showed positive alkaline phosphatase staining. Scale bar: 20 μm. b Five weeks following injection, a 1.5 × 1.5 × 1 cm^3^ size tumor formed. c H&E staining image showed that tumor tissue was noted to be derived from all three embryonic layers obviously, containing glandular epithelium (endoderm), cartilage (mesoderm), and cornified epithelium (ectoderm). Scale bar: 20 μm. (PDF 731 kb)

Additional file 2: **Figure S2.** Quantitative RT-PCR for the expression levels of 13 candidate miRNAs. U6 was used as the internal control for mRNA. Error bars show the SD (*n* = 3). (PDF 565 kb)

## Abbreviations

DEmiRNAs: Differentially expressed miRNAs
EBs: Embryoid Bodies
Gata4: GATA binding protein 4
Gata6: GATA binding protein 6
GCG: Glucagon
Gck: Glucokinase
Glut2: Facilitated glucose transporter, member 2
GO: Gene ontology
H&E: Hematoxylin and eosin
IPC: Insulin-producing cell
iPSC: Induced pluripotent stem cell
ISL: ISL LIM homeobox
KEGG: Kyoto Encyclopedia of Genes and Genomes
Mafa: v-maf musculoaponeurotic fibrosarcoma oncogene family, protein A
miRNA: MicroRNA
MPC: Multilineage precursor stem cell
NC: Negative control
NGN3: Neurogenin 3
Nkx2.2: NK2 homeobox 2
Nkx6.1: NK6 homeobox 1
Pax4: Paired box 4
Pax6: Paired box 6
Pdx1: Pancreatic and duodenal homeobox 1
pGSK3β: Phosphorylated glycogen synthase kinase-3β
qPCR: Real-time quantitative polymerase chain reaction
Sox9: SRY (sex-determining region Y)-box9
SST: Somatostatin
Stat3: Signal transducer and activator of transcription 3
STZ: Streptozotocin
T1D: Type 1 diabetes
UTR: Untranslated region
β-Catenin: Cadherin-associated protein, beta1
