# Supplementary figures and images for: microRNA-690 regulates induced pluripotent stem cells (iPSCs) differentiation into insulin-producing cells by targeting Sox9
Source: Stem Cell Res Ther. 2019 Feb 15;10:59. doi: 10.1186/s13287-019-1154-8 (PMC6376733; doi:10.1186/s13287-019-1154-8)

**a**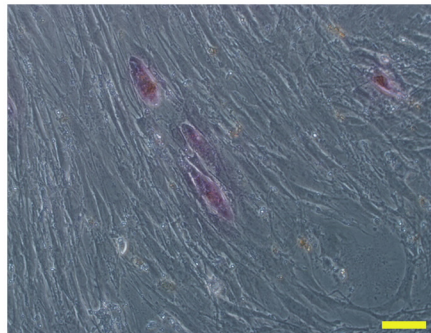**b**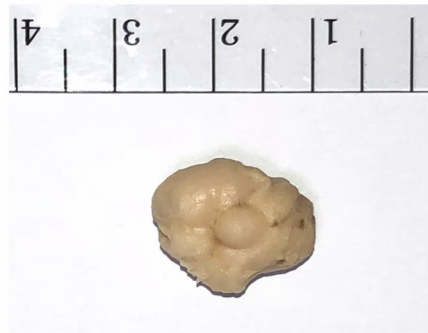**c****Endoderm**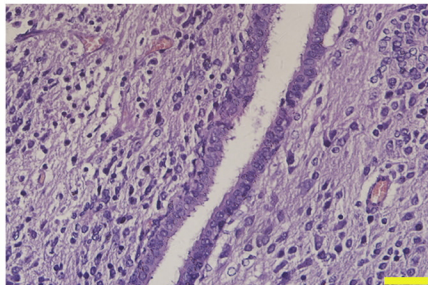**Mesoderm**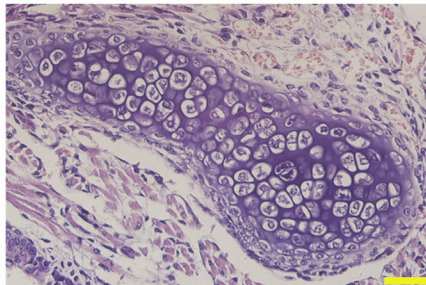**Ectoderm**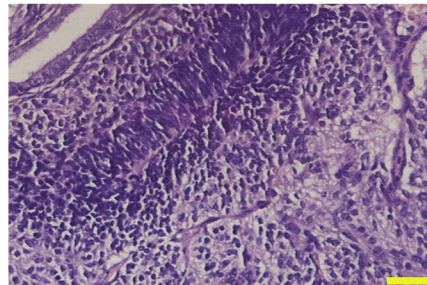

Supplement: Supplementary file 1 — Figure S1. Identification of iPSCs. a The GFP-iPSCs were purple which showed positive alkaline phosphatase staining. Scale bar: 20 μm. b Five weeks following injection, a 1.5 × 1.5 × 1 cm3 size tumor formed. c H&E staining image showed that tumor tissue was noted to be derived from all three embryonic layers obviously, containing glandular epithelium (endoderm), cartilage (mesoderm), and cornified epithelium (ectoderm). Scale bar: 20 μm. (PDF 731 kb) [file 13287_2019_1154_MOESM1_ESM.pdf]

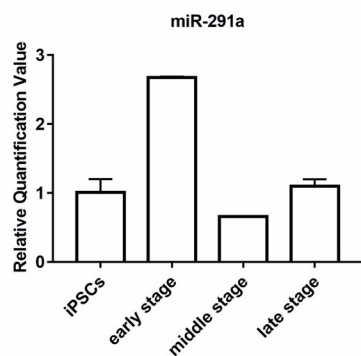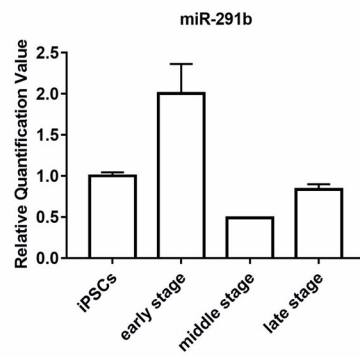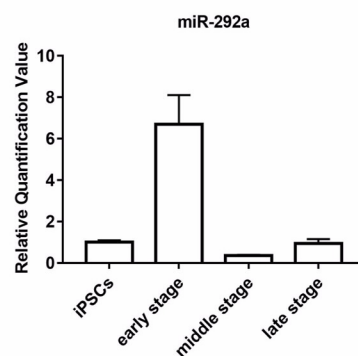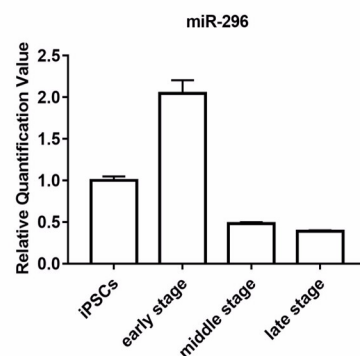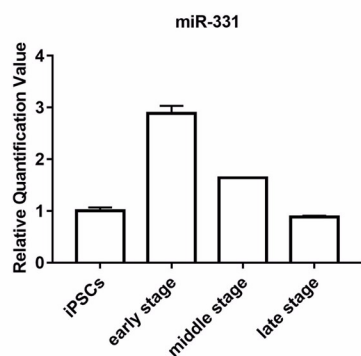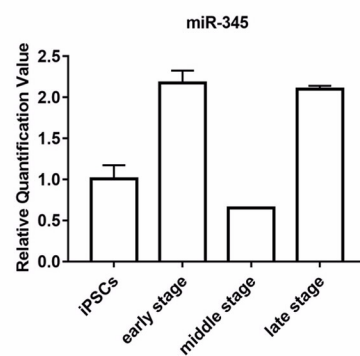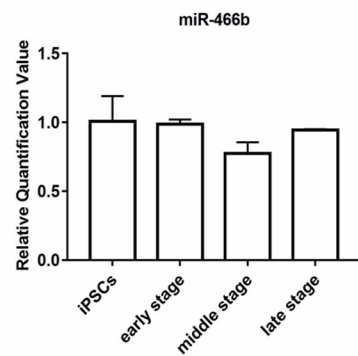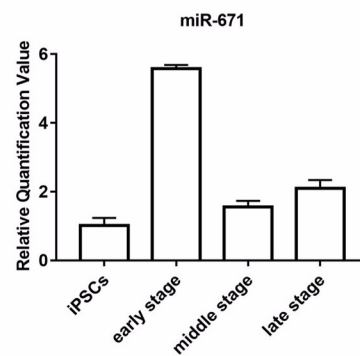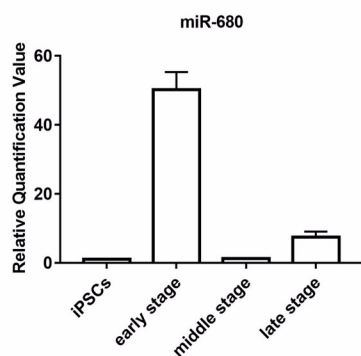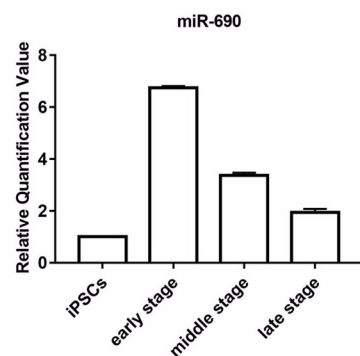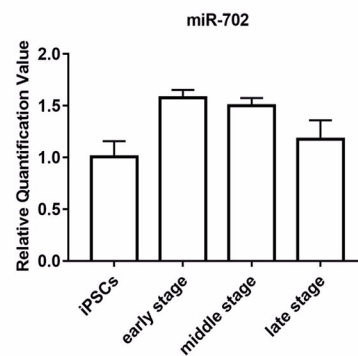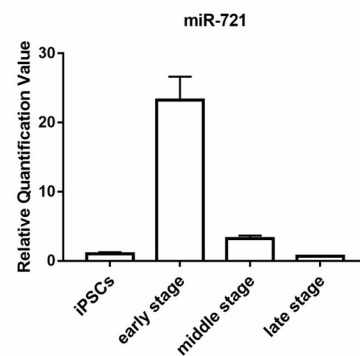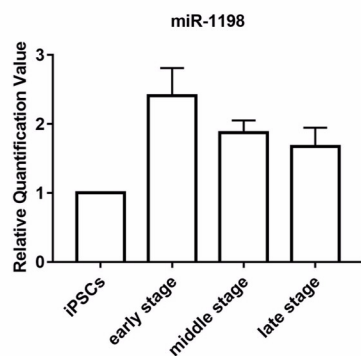

Supplement: Supplementary file 2 — Figure S2. Quantitative RT-PCR for the expression levels of 13 candidate miRNAs. U6 was used as the internal control for mRNA. Error bars show the SD (n = 3). (PDF 565 kb) [file 13287_2019_1154_MOESM2_ESM.pdf]
